# Comparison of the Apoptotic Effects of Topically Applied Papaverine, Diltiazem, and Nitroprusside to Internal Thoracic Artery

**DOI:** 10.21470/1678-9741-2019-0251

**Published:** 2020

**Authors:** Orcun Unal, Mustafa Ozer Ulukan, Vedat Bakuy, Behiye Kaytaz, Sevilhan Artan, Erinc Aral, Didem Melis Oztas, Metin Onur Beyaz, Murat Ugurlucan, Behcet Sevin

**Affiliations:** 1 Department of Cardiovascular Surgery, Eskisehir Osmangazi University Medical Faculty, Eskisehir, Turkey.; 2 Department of Cardiovascular Surgery, Yedikule Hospital of Pulmonary Diseases and Thoracic Surgery, Istanbul, Turkey.; 3 Department of Cardiovascular Surgery, Istanbul Medipol University Medical Faculty, Istanbul, Turkey.; 4 Department of Cardiovascular Surgery, Baskent University Medical Faculty, Ankara, Turkey.; 5 Department of Genetics, Eskisehir Osmangazi University Medical Faculty, Eskisehir, Turkey.; 6 Department of Histology and Embryology, Eskisehir Osmangazi University Medical Faculty, Eskisehir, Turkey.; 7 Cardiovascular Surgery Clinic, Bagcilar Education and Research Hospital, Istanbul, Turkey.

**Keywords:** Mammary Arteries, Vasodilatador Agents, Coronary Artery Bypass, Apoptosis, Diabetes Mellitus, Diltiazem, Biotin, Hematoxylin

## Abstract

**Objective:**

To detect and to compare the apoptotic effects of intraoperatively topically applied diltiazem, papaverine, and nitroprusside.

**Methods:**

Internal thoracic artery segments of ten patients were obtained during coronary bypass grafting surgery. Each internal thoracic artery segment was divided into four pieces and immersed into four different solutions containing separately saline (Group S), diltiazem (Group D), papaverine (Group P), and nitroprusside (Group N). Each segment was examined with both hematoxylin-eosin and the terminal deoxynucleotidyl transferase-mediated dUTP-biotin nick end labeling (TUNEL) method in order to determine and quantify apoptosis.

**Results:**

Apoptotic cells were counted in 50 microscopic areas of each segment. No significant difference was observed among the four groups according to hematoxylin-eosin staining. However, the TUNEL method revealed a significant increase in mean apoptotic cells in the diltiazem group when compared with the other three groups (Group S=4.25±1.4; Group D=13.31±2.8; Group N=9.48±2.09; Group *P*=10.75±2.37). The differences between groups were significant (*P*=0.0001). No difference was observed between the samples of the diabetic and non-diabetic patients in any of the study groups.

**Conclusion:**

The benefit of topically applied vasodilator drugs must outweigh the potential adverse effects. In terms of apoptosis, diltiazem was found to have the most deleterious effects on internal thoracic artery graft segments. Of the analyzed medical agents, nitroprusside was found to have the least apoptotic activity, followed by papaverine. Diabetes did not have significant effect on the occurrence of apoptosis in left internal thoracic artery grafts.

**Table:** 

Abbreviations, acronyms & symbols	
**ANOVA** **CABG** **DM** **LITA** **PBS** **SD** **SPSS** **TUNEL**	**= Analysis of variance** **= Coronary artery bypass grafting** **= Diabetes mellitus** **= Left internal thoracic artery** **= Phosphate buffered saline** **= Standard deviation** **= Statistical Package for the Social Sciences** **= Terminal deoxynucleotidyl transferase-mediated** **dUTP-biotin nick end labeling**

## INTRODUCTION

Coronary artery bypass grafting (CABG) may be performed with the use of arterial and venous grafts^[1]^. The actual aim of CABG is anastomosis of the left internal thoracic artery (LITA) to the left anterior descending artery of the heart, and the other coronary arteries may be revascularized with alternative arterial or venous conduits. The LITA graft is the single most important graft in surgery for its favorable long-term patency compared to other grafts^[2]^. Arterial grafts in contrast to venous grafts have longer patency rates; however, they carry the risk to develop spasm, which may lead to life-threatening conditions^[1]^. The patency of arterial conduits as well as the LITA graft was shown to be enhanced by avoiding graft spasm with the use of vasodilators^[1,3]^. Various pharmacologic vasodilator drugs are shown to prevent spasm and relax the vessel through specific individual mechanisms and there is no single best vasodilator agent or combination of vasodilators to prevent or treat spasm of the arterial grafts^[1]^.

Apoptosis has been considered as an important factor in the development of vascular lesions and atherosclerosis^[4]^. As a result, prevention of apoptosis in LITA grafts is very important for the long-term patency of the graft^[1]^. Apoptosis of endothelia is not only an atherogenic factor itself, but it also regulates apoptosis in vascular smooth muscle cells^[6]^. Turnover rate and apoptosis are low in vascular smooth muscle cells of normal arteries of the adult population^[7]^. Occurrence of apoptosis in vascular smooth muscle cells may have several consequences: (i) these cells are the only cells known to secrete collagen in fibrous capsule, which provides elasticity, and the loss of these cells eventually leads to weakening of the plaque and rupture^[8]^; (ii) the risk of thrombosis increases^[9]^; (iii) plaque calcification occurs through the remnants of apoptotic cells, such as matrix vesicles^[4]^.

Intraoperatively used vasodilators have been analysed for their effects extensively^[1,10-12]^; however, literature lacks *in vitro* comparative analysis for the apoptotic effects of topically used vasodilators. The topical use of papaverine, diltiazem, and nitroprusside during LITA preparation for CABG has been investigated by several authors showing controversial results. We aimed to discuss the effects of these drugs on the development of endothelial apoptosis in LITA grafts immediately after the harvesting maneuvers.

## METHODS

The study was approved by the institutional ethics board of the Eskisehir Osmangazi University Medical Faculty. This was designed as a randomized prospective case control study. Ten consecutive patients electively scheduled for isolated CABG were enrolled to the research. Seven patients were male, and the mean age was 65.5±7.7 (range: 50-80) years. Six patients were diabetic. The patients’ ejection fractions were within normal limits. There were no other significant risk factors in the patients’ preoperative workup. Diabetic patients were operated electively after the blood glucose levels were stabilized.

### Tissue Sampling and Processing

All patients were operated through median sternotomy. After sternotomy, LITA was harvested using low-voltage electrocautery and the side branches were ligated with hemoclips. The harvesting was extended up to the origin of LITA at the left subclavian artery and to the bifurcation site distally. The distal end of LITA graft was transected three minutes after intravenous injection of 300 u/kg unfractionated heparin. Tissue samples 1-1.5 cm long were obtained from the distal ends of the LITA grafts. Each sample was divided into four pieces (2 to 2.5 mm long pieces) using 2.5x magnification loupes and placed into one of the following containers: control (Group S, 10 mL 0.9% NaCl solution at room temperature), diltiazem (Group D, 25 mg diltiazem HCl in 10 mL isotonic solution at room temperature), nitroprusside (Group N, 5 mg nitroprusside sodium in 10 mL 5% dextrose solution at room temperature), and papaverine (Group P, 2 mL 2% papaverine HCl in 10 mL isotonic solution at room temperature). The tissue samples were kept in the containers for 60 minutes, and then they were all transferred into neutral formalin solution (10 mL formalin, 90 mL distilled water, 0.35 g sodium hydrogen phosphate, and 0.656 g disodium hydrogen phosphate) for tissue fixation for 24-48 hours. After tissue fixation, the samples were examined through routine tissue follow-up method; samples were irrigated with tap water for three hours and kept in the following solutions in this order: 70% ethanol for 15 minutes, 80% ethanol for 15 minutes, 90% ethanol for 15 minutes, 96% (I) ethanol for 15 minutes, 96% (II) ethanol for 15 minutes, xylol (I) for three minutes, and xylol (II) for one minute for transparency. Then paraffin blocks were prepared for each sample: paraffin (I) 15 minutes, paraffin (II) 30 minutes, and paraffin (III) 45 minutes for preparing blocks. Thin slices of tissues (5µ thick) were obtained with microtome. Hematoxylin-eosin stains were used to show the structural properties.

In order to determine apoptosis, terminal deoxynucleotidyl transferase-mediated dUTP-biotin nick end labeling (TUNEL) technique was used. Roche Cat. No. 1 684 795 *in situ* cell detection fluorescein kit was used for the TUNEL method. The procedure was performed in the following order: (i) 5µ thick tissue samples were placed on the microscopic slides processed with poly-L-lysine and they were deparaffinized for 45 minutes in xylol; (ii) the tissue slides were irrigated with the following solutions, absolute alcohol, 96% ethanol, 90% ethanol, 80% ethanol, 70% ethanol, and distilled water; (iii) slides were irrigated with phosphate buffered saline (PBS) solution; (iv) permeabilization solution was put on the slides and they were covered with parafilm and left in the incubator at 37 ^(o)^C for eight minutes; (v) slides were irrigated with PBS solution twice; (vi) the marker and enzyme solutions of the study kit were mixed and put on the slides; (vii) slides were covered with parafilm again and they were left in the 37 ^(o)^C incubator for one hour; (viii) the slides were taken into dark area and they were irrigated with PBS solution for three times; (ix) the slides were left in the room temperature for drying; (x) slides were dyed with 4’,6-diamidino-2-phenylindole dye for visualizing the marked cells; (xi) Vectra shield drops were put on the slides and they were covered with covering slide; (xii) and the slides were kept in dark until the time of examination. The solutions used in TUNEL method are (i) 10X PBS solution: 8 g NaCl, 2.01 g KCl, 11.36 g KH_2_PO_4_, 2.04 g Na_2_HPO_4_, 1 liter of distilled water, and HCl (to maintain pH at 7.0); (ii) 1X PBS solution: 20 mL 10X PBS, 180 mL of distilled water, HCl (to maintain pH at 7.0); and (iii) marker solution: 50 µL enzyme solution (vial 1) and 450 µL marker solution (vial 2).

The forty samples obtained from ten patients were assigned into four groups: Group S (isotonic solution, n=10), Group D (diltiazem, n=10), Group N (nitroprusside, n=10), and Group P (papaverine, n=10). All groups were subgrouped according to presence of diabetes mellitus.

### Cell Count

All groups were compared histologically by the slides stained with hematoxylin-eosin. Cell counts were obtained in randomly selected 5-6 slides processed with TUNEL technique and 50 microscopic areas under x100 magnification microscope in order to figure out the differences between the groups. Fluorescein microscopic evaluation was made with the Olympus BX61 fluorescein microscope by the faculty’s genetics and histology departments. Each sample’s apoptotic cell count was derived by taking the average apoptotic cell count from 50 randomly selected areas.

### Statistical Analysis

Statistical analyses were performed with the Statistical Package for the Social Sciences (SPSS) statistics software program (SPSS-IBM, Armonk, New York, United States of America), version 13.0. Discrete variables were expressed in frequencies and percentages and continuous variables as mean±standard deviation (SD). Apoptotic cell counts were compared with one-way analysis of variance (ANOVA) test and the variance homogeneities were determined with Levene’s test. The differences between groups were calculated with post hoc and Tukey’s multiple comparison tests. The diabetic and non-diabetic patients’ samples were compared with Mann-Whitney U test and Monte Carlo comparison where appropriate. A *P*-value < 0.05 is considered statistically significant.

## RESULTS

The general structural properties were examined on the slides stained with hematoxylin-eosin. In Group S, the structural integrity of the endothelial lining of tunica media was found to be preserved. The outer layers, tunica media and tunica adventitia, were also intact. Group S slides did not show histologic differences from each other. Group D slides also showed preserved arterial structure and there were no histologic differences from the other groups. Similarly, histologic analysis of Groups N and P revealed preserved arterial integrity without differences when compared with the other groups. Endothelial lining was intact in all four groups.

**Fig. 1:**
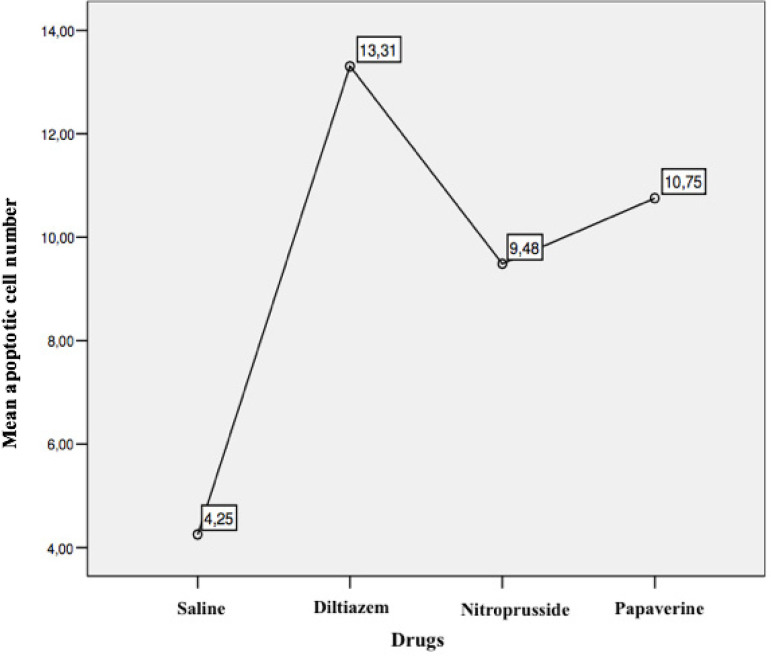
Mean apoptotic cell counts of the groups.

In the fluorescein microscopic analysis of slides processed with the TUNEL method, the lowest number of apoptotic cells was found in Group S slides (4.25±1.4 cells) and the highest number of apoptotic cell count was detected in Group D slides (13.3±2.8 cells). Group N (9.48±2.09 cells) and Group P (10.75±2.37 cells) slides showed moderate numbers of apoptotic cells. The number of apoptotic cells in each group are presented on Table 1 and Figure 1. Group S slides showed scarce presence of apoptosis (Figure 2A, Table 1). None of the 100 scanned areas of ten samples had more than nine apoptotic cells. Group D slides showed intense apoptosis (Figure 2B, Table 1). Apoptosis was profoundly evident in all three layers of the arterial samples including tunica intima, media, and adventitia in Group D. We also detected apoptosis in each layer of the arterial samples in Group N (Figure 2C, Table 1) and Group P (Figure 2D, Table 1); however, the values were lower than the amounts detected in Group D slides.

**Fig. 2A:**
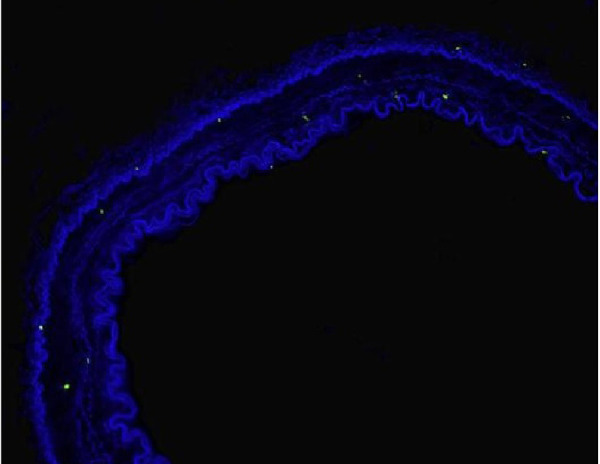
Fluorescein microscopy of slides processed with the terminal deoxynucleotidyl transferase-mediated dUTP-biotin nick end labeling (TUNEL) method in the isotonic solution group (Group S).

**Fig. 2B:**
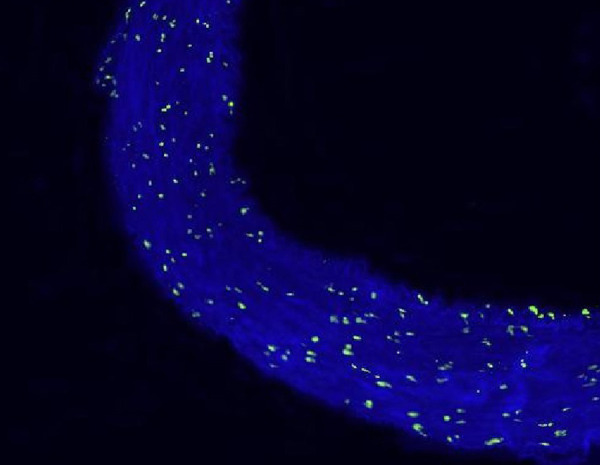
Terminal deoxynucleotidyl transferase-mediated dUTPbiotin nick end labeling (TUNEL) stain of an artery in the diltiazem group (Group D).

**Fig. 2C:**
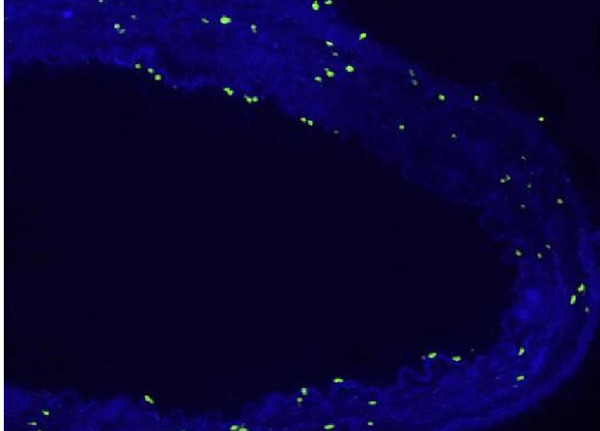
Terminal deoxynucleotidyl transferase-mediated dUTP-biotin nick end labeling (TUNEL) stain of an artery in the nitroprusside group (Group N).

**Fig. 2D:**
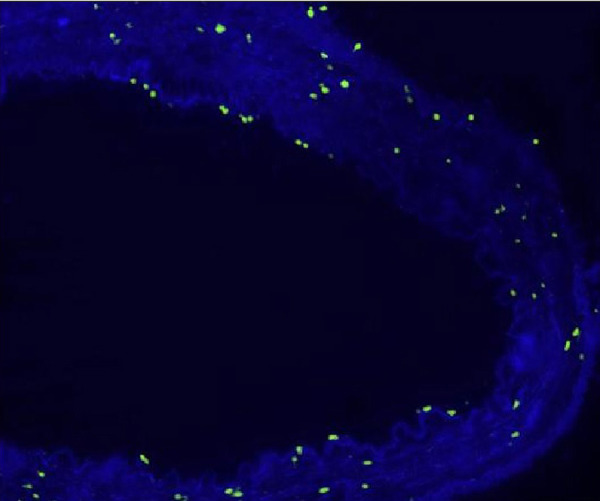
Terminal deoxynucleotidyl transferase-mediated dUTPbiotin nick end labeling (TUNEL) stain of an artery in the papaverine group (Group P).

**Table 1:** Number of apoptotic cells in different groups.

Descriptives										
Number of counted cells										
		**N**	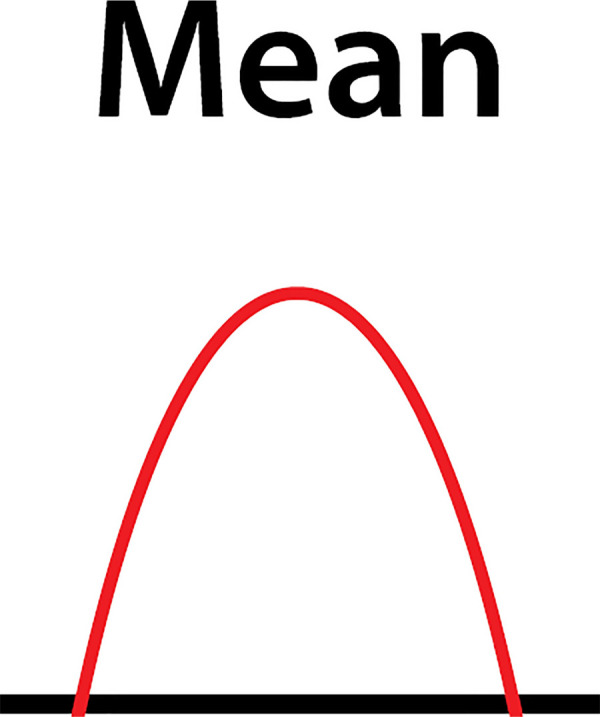	**Standard** **deviation**	**Standard** **error**	**95% Confidence** **interval for mean**		**Minimum**	**Maximum**	**Between-component variance**
						**Lower** **bound**	**Upper** **bound**			
Isotonic solution		500	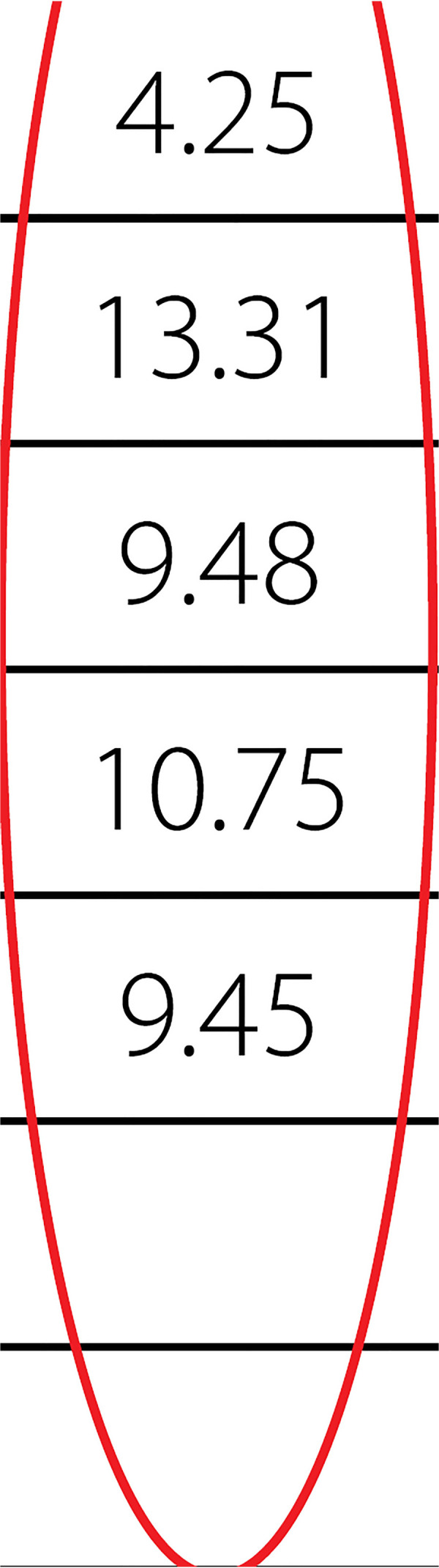	1.401	0.063	4.13	4.37	1	9	
Diltiazem		500		2.819	0.126	13.06	13.55	6	22	
Nitroprusside		500		2.093	0.094	9.30	9.67	4	18	
Papaverine		500		2.372	0.106	10.55	10.96	4	20	
Total		2000		3.985	0.089	9.27	9.62	1	22	
Model	Fixed effects			2.232	0.050	9.35	9.55			
	Random effects				1.906	3.38	15.52			14.527

Comparison of the average apoptotic cell counts between the groups are presented on Figure 1. The comparisons revealed that Group D has the highest number of apoptotic cells (Table 1). In descending order, Group D was followed by Group N, Group P, and Group S. The four groups’ variances were found to be homogeneous at Levene’s test (*P*=0.061) and ANOVA test revealed statistically significant differences between the groups (*P*=0.0001). Post hoc test and Tukey’s multiple comparisons performed after ANOVA test resulted in statistically significant difference between all groups (Table 2). The biggest difference was between Groups S and D (-9.056), whereas the difference between Groups N and P was the lowest (-1.27).

**Table 2:** Comparison between the groups with post hoc and Tukey's tests.

Post Hoc Tests						
Multiple comparisons						
Dependent variable: number of counted cells						
Tukey honestly significant difference test						
**(I) Agent**	**(J) Agents**	**Mean difference (I-J)**	**Standard error**	**Significance**	**95% Confidence interval**	
					**Lower bound**	**Upper bound**
Isotonic solution	Diltiazem	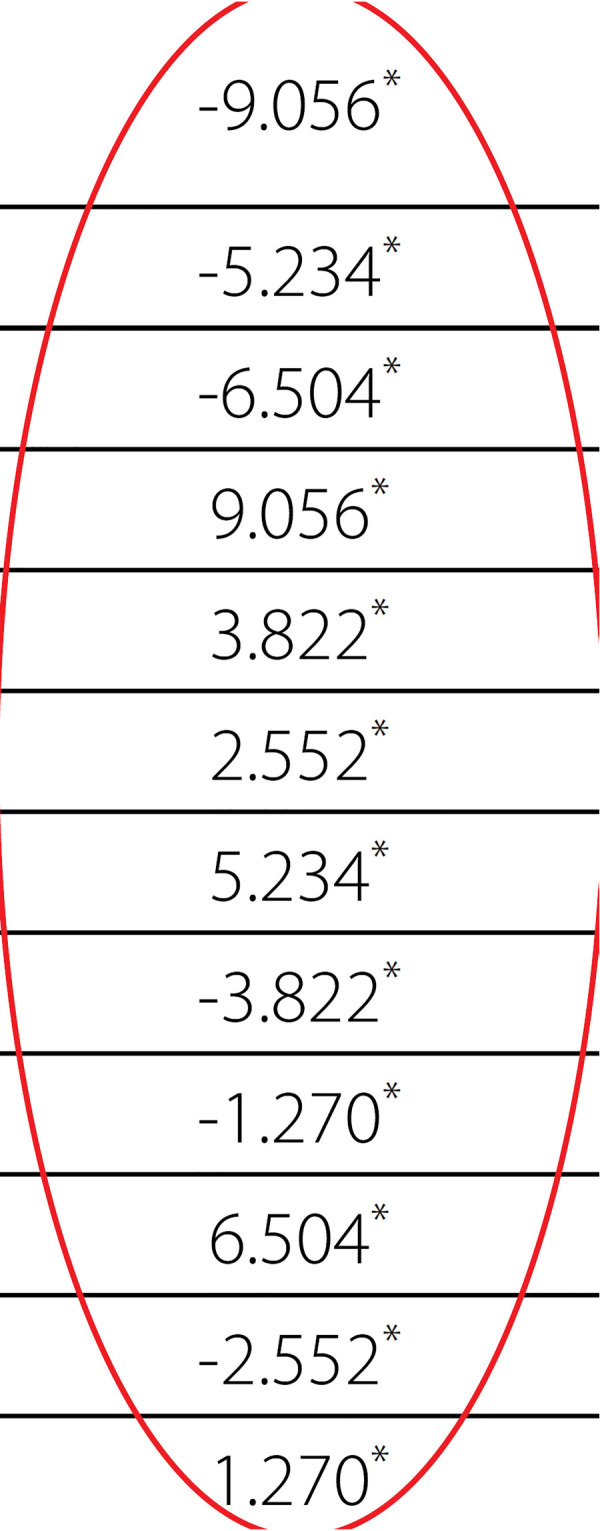	0.141	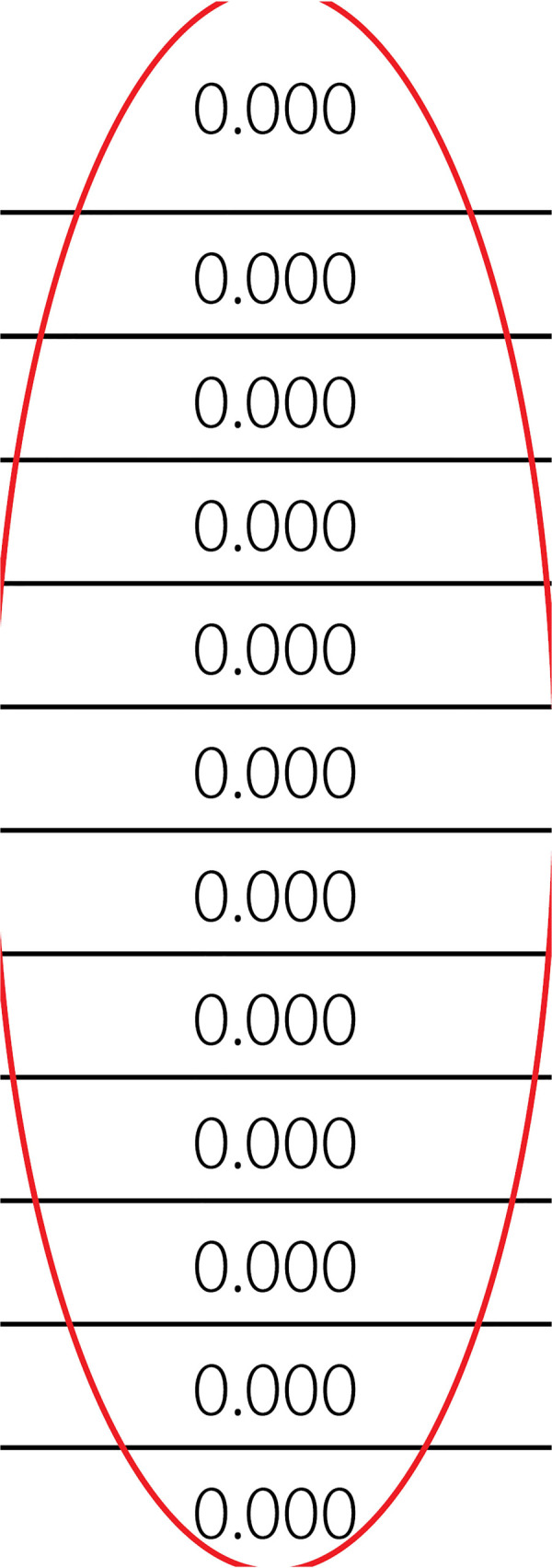	-9.42	-8.69
	Nitroprusside		0.141		-5.60	-4.87
	Papaverine		0.141		-6.87	-6.14
Diltiazem	Isotonic solution		0.141		8.69	9.42
	Nitroprusside		0.141		3.46	4.18
	Papaverine		0.141		2.19	2.91
Nitroprusside	Isotonic solution		0.141		4.87	5.60
	Diltiazem		0.141		-4.18	-3.46
	Papaverine		0.141		-1.63	-.91
Papaverine	Isotonic solution		0.141		6.14	6.87
	Diltiazem		0.141		-2.91	-2.19
	Nitroprusside		0.141		0.91	1.63

*The mean difference is significant at the 0.05 level

Comparisons between diabetic and non-diabetic subgroups were made with Mann-Whitney U test and Fisher’s exact test where appropriate (Table 3) and the analyses did not show any significant difference between these subgroups in terms of apoptotic cell counts.

**Table 3:** Comparison between diabetic and non-diabetic patients' samples within groups.

Groups	Glycemic status	Mean*±SD (min-max)	P1	P2
Group S (n=10)	DM (+) (n=6)	4.23±1.90 (3.99-4.53)	0.748	0.799
	DM (-) (n=4)	4.26±2.30 (3.99-4.53)		
Group D (n=10)	DM (+) (n=6)	13.07±0.49 (12.40-13.76)	0.394	0.476
	DM (-) (n=4)	13.20±0.82 (11.97-13.74)		
Group N (n=10)	DM (+) (n=6)	9.30±0.56 (8.34-9.84)	0.201	0.255
	DM (-) (n=4)	9.59±0.13 (9.47-9.71)		
Group P (n=10)	DM (+) (n=6)	10.80±0.46 (10.06-11.38)	0.201	0.255
	DM (-) (n=4)	10.64±0.04 (10.61-10.70)		

P1=Mann-Whitney U test; P2=Monte Carlo test; DM=diabetes mellitus; SD=standard deviation

* Mean number of apoptotic cells

## DISCUSSION

CABG is still the gold standard treatment option for the revascularization of ischemic myocardium with long-term established graft patency rates. The realistic aim of CABG is the anastomosis of LITA to the left anterior descending coronary artery. Additional grafts may be used for the revascularization of the other coronary vessels. It is well known that arterial conduits have better patency rates when compared with the venous grafts; however, arterial grafts, including LITA, poses the risk of vasospasm. Vasospasm may be minimized with meticulous surgical techniques in combination with pharmacologic agents. Modern pharmacology offers various vasodilator agents with clearly defined mechanism of actions. However, profound superiority of one agent over another has not been proven. Literature lacks an established one single agent or combination therapy to overcome the spasm in arterial conduits^[1,13]^. In addition, the vasodilator medication, when applied, should carry properties to prevent or minimize apoptosis for the long-term patency of the grafts.

The main finding of this study is the confirmation of occurrence of apoptosis in LITA grafts and we quantified the apoptotic effects of the topically applied vasodilators. It is clear that all vasodilators have apoptotic effects to some extent; however, diltiazem was found to cause more apoptosis whereas nitroprusside had the lowest apoptotic effect. The differences between all agents were found to be statistically significant in the current research.

Apoptosis of smooth muscle cells in saphenous vein grafts have been reported before^[14-16]^ and it was attributed to the grafts’ mechanical stretch. Kocailik et al.^[16]^ studied effects of papaverine on the cell viability of saphenous vein segments. They concluded that papaverine improved endothelial cell viability and was associated with preservation of the maximal endothelial-dependent vasodilator response of the saphenous vein graft segment^[16]^. In contrast, Gao et al. showed the apoptotic effects of papaverine in details^[17,18]^ and they proposed the use of less apoptotic agents. Other than papaverine, there are some commonly used vasodilators, such as diltiazem and nitroprusside, and their apoptotic effects need to be studied in detail and compared in the literature.

Papaverine is one of the most commonly used topical vasodilators in CABG surgery^[19]^. It is well known that it increases LITA flow when applied topically or injected intraluminally^[20]^. Papaverine has been reported to cause apoptosis in all three layers of the arterial wall^[17]^. An important aspect of papaverine is that it can only be dissolved in acidic environment. Although it was accused to cause structural and functional damage by means of acidity^[21]^, a controlled comparison with similar acidic solutions did not show endothelial or smooth muscle cell apoptosis^[18]^. Moreover, bovine aorta apoptosis was found to be attenuated by papaverine^[22]^. Another probable mechanism of apoptosis induced by papaverine may be via increased intracellular cyclic adenosine monophosphate^[23]^. Thirdly, calcium antagonist effect of papaverine may play a role in triggering apoptosis^[24]^. Mitochondrial dysfunction caused by papaverine has also been proposed as a probable mechanism of apoptosis^[25]^. The histologic results of our study confirmed those findings; however, the amount of apoptosis with papaverine was higher than with isotonic solution and nitroprusside, and it was found to be lower than with diltiazem in our study.

Diltiazem is a calcium channel blocker which acts through L-type channel blockage^[1,26]^. It is another commonly applied vasodilator agent to prevent vasospasm and increase flow of coronary artery bypass conduits during CABG. Intraoperative intravenous infusion of diltiazem in addition to topical application of papaverine to test LITA flow was assessed by Erdem O et al.^[27]^, and the authors found that the maneuver significantly increased the arterial graft flows as well as provided lower incidence of atrial fibrillation during the intraoperative and postoperative periods^[27]^. Other than the flow alteration properties of diltiazem, apoptotic properties of this agent on tissues were also assessed in the literature. Occurrence of apoptosis was reported by calcium channel blockers in different studies^[23]^, but the exact mechanisms are unknown. On the other hand, calcium channel blockers have been reported to attenuate apoptosis in different cells, including human aortic cell cultures^[28]^. The apoptotic effects of calcium-channel blockers were reported to be both dependent and independent from calcium^[23,29]^. Another probable pathway for apoptosis induced by these drugs may be the intracellular kinases and membrane transport proteins^[29]^. Diltiazem caused apoptosis more than the other agents in our study. This apoptotic effect may counteract the vasodilatory effects of the drug.

Sodium nitroprusside is a potent vasodilator which is clinically used very frequently in hypertensive patients. It leads to vasodilation via its active metabolite nitric oxide, by causing relaxation in smooth muscle cells^[1]^. Cooper et al.^[30]^ compared saline, papaverine, nifedipine, glyceryl trinitrate, and sodium nitroprusside and measured the flow rates of LITA grafts prepared for CABG in their study. They observed that sodium nitroprusside revealed fourfold increase in LITA flow rates when it was flushed over the conduit^[30]^. Similar finding was observed by Tezcaner et al.^[31]^ in their study when they compared papaverine with sodium nitroprusside. It is known that nitroprusside had both apoptotic effects on vascular smooth muscle cells and antiapoptotic effects on vascular endothelia^[32]^. Nitroprusside has been hypothesized to reveal apoptosis through both cyclic guanosine monophosphate-dependent and -independent pathways^[33,34]^. The comparison of nitroprusside indicated that the drug caused less apoptosis than papaverine and diltiazem, but more apoptosis than isotonic solution in our study. As a result, as a less apoptotic agent, nitroprusside may be preferred over the other vasodilators; however, the long-term results remain to be determined.

Despite the effects of vasodilator agents on LITA grafts, Ozkara et al.^[5]^ in their study indicated that the LITA grafts did not require additional vasodilator treatment when they were harvested with no-touch technique with a wide pedicle and were trimmed more than 2 cm proximal to the bifurcation. Even if the blood flow was still low with careful harvesting measures, the authors proposed to wait until the spontaneous recovery of the spasm. Otherwise, vasodilator therapy with nitroprusside could have been helpful; however, hypotension is a side effect of this agent and could be hazardous in certain cases^[5]^.

Another issue discussed in the current research is the comparison between diabetic and non-diabetic patients. Some authors postulated that apoptosis occurred more frequently in endothelia, which were exposed to high glucose concentrations^[35,36]^. However, in our study, the results were not in accordance with those reports. In all groups, including the control group, the amount of apoptosis was not significantly different between the diabetic and non-diabetic patients’ samples. Hence, we may consider that amount of apoptosis in LITA grafts does not differ in diabetic and non-diabetic patients.

### Limitation

This study has certain limitations. The small number of samples is one of the major limitations. Another major limitation accounts for the *in vitro* design of the research. The possible implications of the study’s translation to clinical applications and long-term follow-up results are warranted in order to determine the best vasodilator agent(s) for the longevity of LITA grafts. The vasodilator agents are solely used in the current study; however, combinational use of these drugs is common. Literature lacks an established single medication or combination regime and each clinic follows its own protocol. Lack of various combinational tests of vasodilators may be accounted as another limitation of the current research.

## CONCLUSION

In conclusion, one of the primary factors in long-term success of CABG operations is the favorable long-term patency rates of LITA grafts. The widely used vasodilator drugs are important to protect the graft from vasospasm. The benefit of these drugs must outweigh the potential adverse effects. Vascular smooth muscle and endothelial cells are important vasoregulators, and their functions may be altered when apoptosis occurs. In terms of apoptosis, diltiazem was found to have the most deleterious effects on LITA grafts. Of the analyzed drugs, nitroprusside was found to have the least apoptotic activity, followed by papaverine. All three agents were found to be more apoptotic than control isotonic solution. Diabetes did not have significant effect on occurrence of apoptosis in LITA grafts in our study.

**Table:** 

Authors' roles & responsibilities	
OU MOU VB BK SA EA DMO MOB MU BS	Substantial contributions to the conception or design of the work; or the acquisition, analysis, or interpretation of data for the work; drafting the work or revising it critically for important intellectual content; final approval of the version to be published Substantial contributions to the conception or design of the work; final approval of the version to be published Substantial contributions to the conception or design of the work; or the acquisition, analysis, or interpretation of data for the work; final approval of the version to be published Substantial contributions to the conception or design of the work; or the acquisition, analysis, or interpretation of data for the work; final approval of the version to be published Substantial contributions to the conception or design of the work; or the acquisition, analysis, or interpretation of data for the work; final approval of the version to be published Substantial contributions to the conception or design of the work; or the acquisition, analysis, or interpretation of data for the work; final approval of the version to be published Substantial contributions to the conception or design of the work; final approval of the version to be published Substantial contributions to the conception or design of the work; final approval of the version to be published Substantial contributions to the conception or design of the work; final approval of the version to be published Substantial contributions to the conception or design of the work; or the acquisition, analysis, or interpretation of data for the work; final approval of the version to be published
